# A numerical investigation of changes in lens shape during accommodation

**DOI:** 10.1038/s41598-021-89145-z

**Published:** 2021-05-05

**Authors:** I. Cabeza-Gil, J. Grasa, B. Calvo

**Affiliations:** 1 Aragón Institute of Engineering Research (i3A), University of Zaragoza, Mariano Esquillor s/n, Zaragoza, 50018 Spain; 2 Centro de Investigación Biomédica en Red en Bioingenieria, Biomateriales y Nanomedicina (CIBER-BBN), Zaragoza, Spain

**Keywords:** Applied optics, Computational biophysics, Biomedical engineering

## Abstract

The purpose of this study was to investigate how the mechanical properties and geometry of the lens influence the changes in lens shape during accommodation. To do so, ex vivo stretching tests of the isolated lens were simulated via finite element analysis. In these tests, the lens is stretched from the accommodated state to the non-accommodated state. Several key characteristics of the lens were studied: the stiffness gradient of the lens material, the distribution of the capsule thickness, the mechanical properties of the capsule and the material comprising the lens, nucleus and cortex, and the influence of two different age-related lens geometries (17 and 29 y/o subjects). To determine the effects on the changes in lens shape during accommodation, changes in the anterior and posterior radius, the lens and nucleus thicknesses and the equatorial lens diameter were analysed. The results suggest that multiple factors exert statistically significant influences on how the lens changes its shape, but two factors predominate over the rest: the stiffness ratio between the nucleus and cortex and the stiffness of the capsule, specifically the posterior surface.

## Introduction

The restoration of dynamic accommodation in presbyopic eyes and in the context of cataracts has not yet been successfully achieved^1^. So far, the most widespread solution has been the use of accommodating intraocular lenses; however, their development remains in progress as the restoration of dynamic accommodation is not yet fully achieved^2,3^. One of the main difficulties in the development of novel ophthalmologic solutions to restore dynamic accommodation is the absence of quality explanations of the biomechanical process of accommodation. On this basis, we hypothesise that numerical methods could help the research community and manufacturers to find better solutions by providing fundamental knowledge of this phenomena.

To the best of our knowledge, several authors have designed finite element (FE) models to explain and understand some aspects of the accommodation process following Helmholtz’s accommodation theory. Burd et al.^4^ designed an axisymmetric model for different ages in an attempt to observe some of the features of presbyopia. Other authors followed the same criteria, using 3D modelling to measure the forces applied by the zonules and the focal changes of the lens^5–7^. Lanchares et al.^8^ reproduced the compliance of the materials by a hyperelastic model, and recently, Wang et al.^9–11^ studied both the focal changes of the lens for different age-related properties and the effects of zonular union.

In more detail, those FE models tried to replicate isolated ex vivo stretching tests of the crystalline lens^12–16^. These experimental protocol tests were performed on post-mortem human and monkey lenses and reported the dynamic optical and biometric changes of the lenses.

The dynamic changes in the anterior and posterior radii of curvature, lens thickness and lens diameter vary slightly upon accommodation depending on the type of hominid and the age of the subject^13,14,17,18^. Therefore, our hypothesis is that the main dynamic biometric changes in hominids depend on lens material properties and geometry, both of which in turn depend on age.

For this reason, to achieve a better understanding of the focal change of the lens during accommodation, the purpose of this study is to shed light on how the lens changes its shape depending on the lens mechanical properties and lens geometry for young subjects using finite element analysis (FEA). To do so, the effects of the mechanical properties of the capsule and the material comprising the lens, nucleus and cortex, on the changes in lens shape were evaluated together with two age-related geometries. Contrary to other FE studies, we attempted to completely describe the changes in lens shape by measuring the changes in anterior and posterior radii, lens and nucleus thicknesses and the equatorial lens diameter.

Due to its complexity, this study also helps to identify the lens material properties in order to determine them experimentally. Burd et al.^19^ reported that the spinning lens measurements of Fischer^20^ might not be reliable, and since then, few studies pertaining to the internal lens mechanical properties have been performed without individually characterising the mechanical properties of the lens nucleus and cortex^21,22,23^.

To provide a more accurate approach, the effects of two key lens mechanical aspects on accommodation were previously studied. On the one hand, the lens stiffness gradient was analysed because there is an evidence that the morphological shape of the lens forms a gradient of refractive index (GRIN) and stiffness^22–25^. On the other hand, the distribution of the capsule thickness was studied as the lens capsule thickness varies along the lens location^26,27^.

The paper is organised as follows. First, the FE model, together the stretching test, is described in “Methods”; then, the case studies analysed in this research are explained. After that, the results are presented sequently as in “Case studies” section to be finally discussed in the last section.

## Methods

The lens geometries of 17 and 29 y/o subjects, the material approach for each tissue and the mesh structure are described in “FE model” section. Then, the lens stretching test simulated in this study is presented in “Analysis procedure” section. Lastly, the case studies are described, which includes the influences of the lens stiffness gradient and the distribution of the capsule thickness on the ability of the lens to change its shape. Both studies were initially performed for the 29 y/o lens geometry. Once both effects were numerically analysed, the lens mechanical properties for two different geometries corresponding to two different ages (17 and 29 y/o) were analysed following the design of experiments (DoE) methodology with a full factorial design^28^.

### FE model

**Figure 1:**
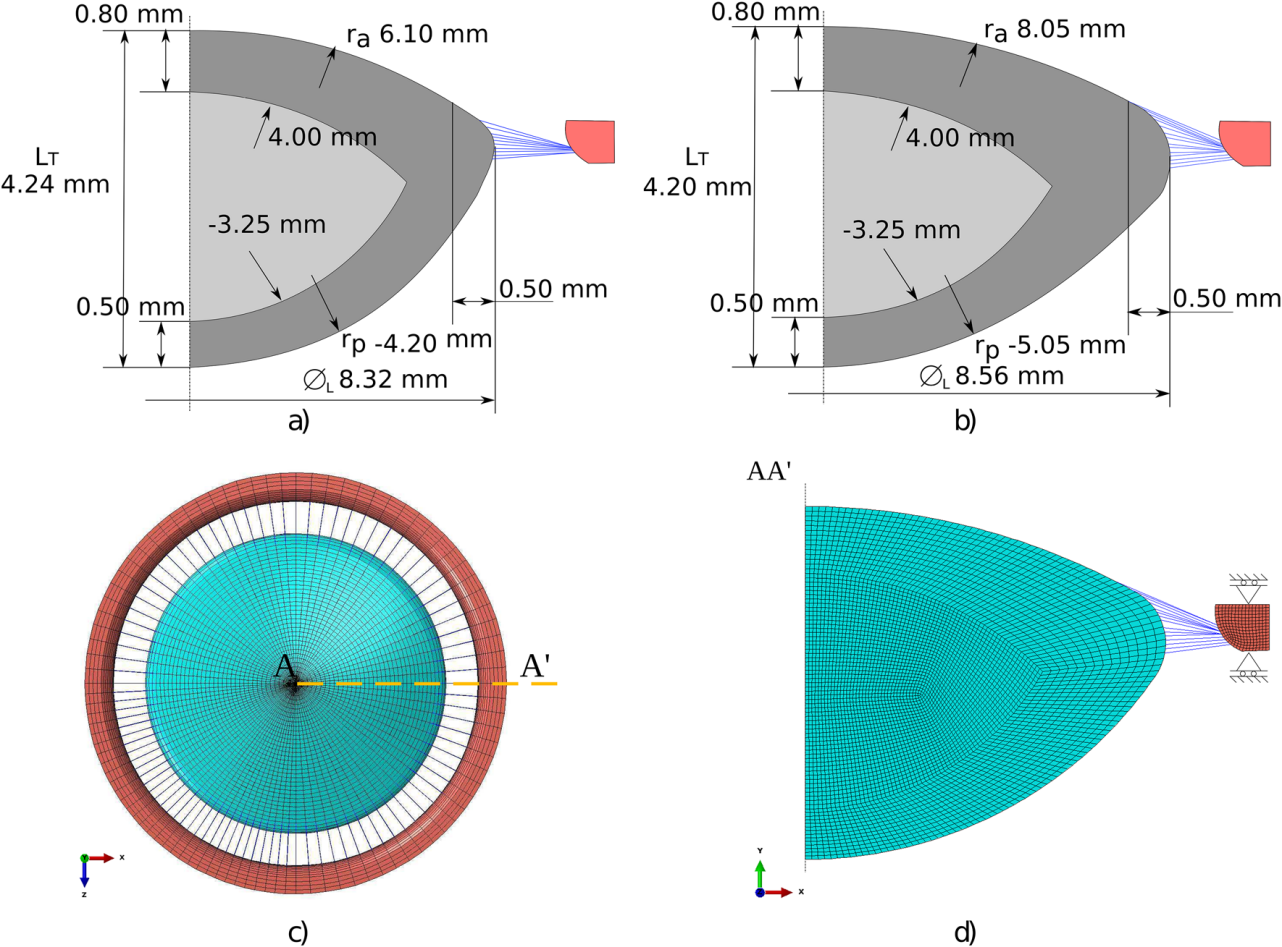
The dimensions of the 17-year-old (**a**) and 29-year-old (**b**) lens geometry. The nucleus is depicted in light grey, whilst the cortex is in dark grey. A plane (**c**) and profile (**d**) view of the FE model for the 29 y/o subject is presented. Zonules and part of the ciliary body ring are depicted in blue and red, respectively.

The lens geometries were generated according to various studies for isolated lenses as a function of age. The anterior and posterior curvature radii were extracted from Borja et al.^29^, whilst the lens thickness and diameter were based on the study by Martinez-Enriquez et al.^30^ For both geometries, the anterior and posterior thickness values of the cortex were 0.80 mm and 0.50 mm, respectively, assuming that hardly any change in the cortex thickness occurs upon accommodation^31,32^. Radii of curvature of the anterior and posterior surfaces of 4.00 and 3.25 mm, respectively, were considered^32,33^. Zonules were anchored 0.50 mm in the anterior capsule and in the lens equatorial diameter according to the study by Bernal et al.^34^. The zonules emerged from the apex area of the ciliary body. Figure 1a,b summarises the lens dimensions for both 17 and 29 y/o subjects.

Abaqus v.14.1 was chosen as the software suite for the FEA. An axisymmetric FE model was designed to perform the lens stretching tests. A mesh sensitivity analysis was previously performed in order to establish the final mesh size; see Fig. 1c,d. The lens nucleus and cortex were considered solid bodies and were meshed with 4672 4-node bilinear axisymmetric quadrilateral hybrid elements (CAX4H); the capsule was meshed with 203 2-node linear axisymmetric membrane elements (MAX1); 10 zonules were modelled using Abaqus connector elements; and the ciliary body was meshed with 231 CAX4H elements. The lens material was modelled with an incompressible linear elastic behaviour (*E*=*f*(*r*, *z*), $\nu =0.5$). The lens capsule was modelled via membrane elements, assuming a state of tensile stress without allowing any bending or transverse shear stiffness. In turn, the mechanical properties of the anterior and posterior lens capsule were modelled individually according to the study of Krag and Andreassen^35,36^, who reported different mechanical properties for both surfaces.

The zonules were modelled with linear connector elements. These elements were characterised by means of a force-displacement equation:

1 $$\begin{aligned} F(u) = k u \text { ,} \end{aligned}$$

with $k= 100$ mN/mm and *u* representing the displacement in the direction of the zonule (element)^37^. No compression was considered.

### Analysis procedure

The experimental lens stretching tests^13,14^ were simulated via FEA. These tests attempt to reproduce the relaxation of the ciliary muscle, adjusting the focal length from the near to the far vision state. Therefore, the reference configuration of our FE model corresponds to the accommodated geometry (near state vision), where a stress-free state of both zonules and lens can be considered. To simulate the relaxation of the ciliary muscle, in all cases of the study, a radial displacement of 0.50 mm in the whole ciliary body ring was imposed, isolating the effect of the stretching process in the lens; see Fig. 1d.

During the simulation, all dynamic optical and biometric lens measurements were evaluated in each time increment. For that purpose, the displacements of the nodes of the lens contour were registered by an URDFIL Abaqus subroutine and post-processed with MATLAB R2020b. To measure the lens power, the thick lens formula was used:

2 $$\begin{aligned} L_P= (n_L - n_{a})\left[ \frac{1}{r_a} - \frac{1}{r_p} + \frac{L_T (n_L - n_{a})}{r_p r_{a} n_L}\right] \text { ,} \end{aligned}$$

where $n_{a} = 1.336$ represents the refractive index of the aqueous humour and $n_L = 1.42$ is the estimated equivalent refractive index of the lens^25^. The remaining biometric terms, $r_a$, $r_p$ and $L_T$, defined in Fig. 1a,b, vary throughout the simulation. The radii of curvature throughout the stretching process were calculated through an equation for a conic section with apex at the origin and tangent to the y-axis.

3 $$\begin{aligned} y^2 - 2rx + (k+1)x^2 = 0 \text { .} \end{aligned}$$

To obtain the corresponding radius (*r*) and the conic constant (*K*), a nonlinear system of equations formed by the coordinates of the nodes (*x*, *y*) was solved. The goodness of the fit was confirmed, with $R^2>99\%$. Note that the change in the lens power ($\Delta L_P$) obtained is negative because the lens power decreases with this stretching process.

### Case studies

#### Evaluation of the lens stiffness gradient

The lens nucleus and cortex are widely modelled in the literature with homogeneous material behaviour^4,7,8^. However, it has been reported that the stiffness varies within the lens according to its morphological shape^21,22^. Weeber et al.^22^ reported that the lens shear modulus (*G*) varies with location, showing a maximum value at a distance of 2.5–3.0 mm from the centre of the lens in young eyes. Weeber et al.^6^ modelled three different cases of age-related lenses with stiffness gradients; however, the mechanical influence of having or not having a stiffness gradient was not analysed. Thus, to evaluate whether the stiffness gradient ($\mathbf {\nabla } E (r,z)$, with *E*(*r*, *z*) representing the elastic modulus) has a significant mechanical influence on the ability of the lens to change its shape, an exhaustive analysis with multiple scenarios of the stiffness gradient in the lens was performed.

**Figure 2:**
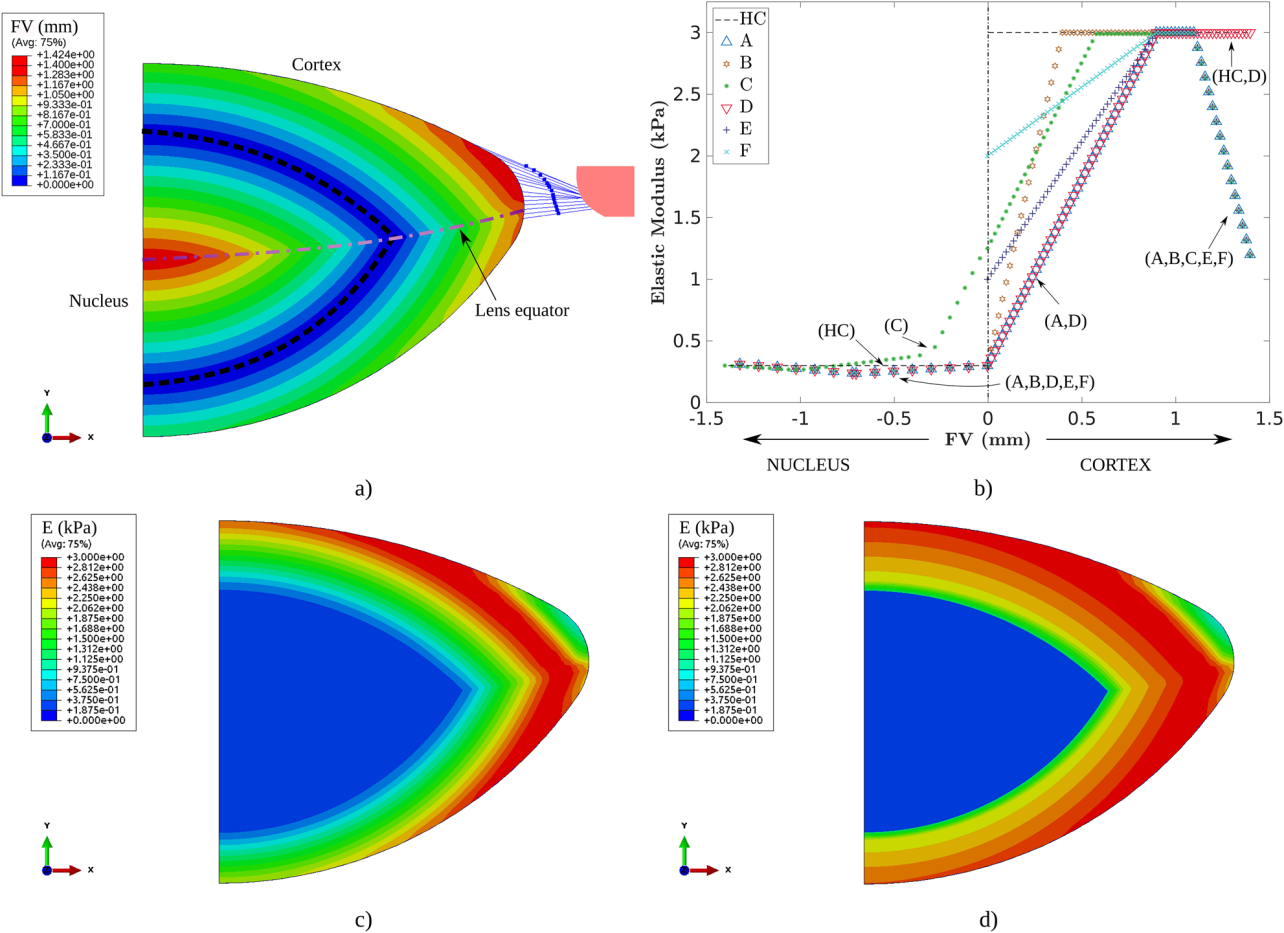
(**a**) FV variable which measures the absolute perpendicular distance from a point in the lens to the anterior or posterior radius of the lens nucleus. (**b**) $E=f(FV)$ for the seven different scenarios analysed. The values of elastic modulus *E* within the lens for scenarios #A and #F are depicted in (**c**) and (**d**), respectively.

To do so, we designed a stiffness gradient ($E=f(FV)$) as a function of the location, creating a field variable *FV* to attempt to reproduce the actual lens layered structure^38^; see Fig. 2a. *FV* measures the perpendicular absolute distance (mm) between a point in the lens and the anterior or posterior radius of the lens nucleus according to the region in which the point is located (anterior or posterior, separated by the lens equator). As observed in Fig. 2a,b, *FV* is 0.00 mm in the anterior and posterior radii of the lens nucleus and reaches its maximum of 1.40 mm in the equatorial lens diameter and in the centre of the lens.

**Figure 3:**
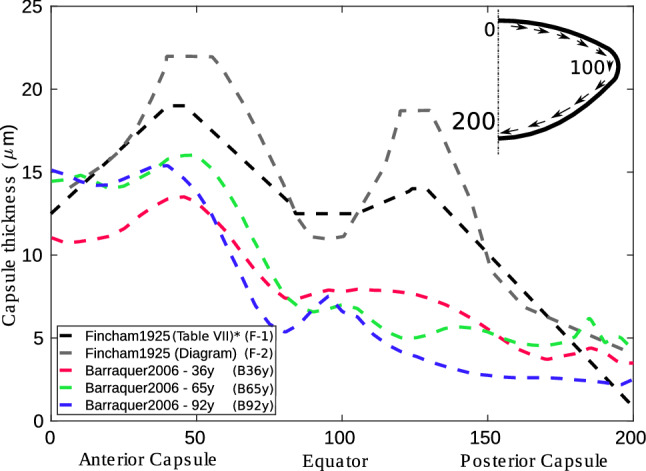
Five different cases of capsule thickness distribution ($\upmu \text{m}$) as a function of the standardised capsule perimeter. An outline for understanding standardisation is attached. *The mean values were obtained with the first and third row data from Table VII—thickness of lens capsule from Fincham^26^. The second row data of this table were discarded due to notable differences with the other data.

Based on the studies by Weebet et al.^22^ and Wilde et al.^23^, and considering that the stress–strain relationship $E=3G$,*G*,$E_{cortex}= 3.00$ kPa and $E_{nucleus}= 0.30$ kPa were calculated as a reference scenario^23^; see case #HC in Fig. 2,$E=f(FV)$. The first gradient (#A) was derived from Weeber’s^22^ measurements for a 30 y/o subject (scaled to fit scenario #HC); see Fig. 2,$E=3.00$,^22^,$FV > 1.10$,^20,31^,2,

In all analyses, the elastic modulus of the anterior capsule was $E= 1.00$ MPa, and that of the posterior capsule was $E=0.70$ MPa. The distribution of capsule thickness reported by Fincham^26^ is shown by the black line in Fig. 3, where the 29 y/o lens geometry was considered.

#### Evaluation of the distribution of the lens capsule thickness

Fincham^26^ and Barraquer et al.^27^ reported five different distributions of the capsule thickness. However, the mechanical influence of this factor has not yet been numerically evaluated. For this reason, five experimental distributions of the capsule thickness were analysed: two of them are reported by Fincham^26^ and indicated by the black and grey dashed lines in Fig. 3, and three different distributions according to different ages of 36, 65 and 92 years are reported by Barraquer et al.^27^. For this study, the elastic modulus of the anterior capsule was 1.00 MPa, and that of the posterior capsule was 0.70 MPa. The nucleus and cortex were considered homogeneous, with $E_{cortex}=3.00$ kPa and $E_{nucleus}=0.30$ kPa. All analyses were calculated for the 29 y/o lens geometry.

Moreover, to help extract and isolate the effects of the lens capsule on the focal changes of the lens, three different uniform thicknesses of 7, 13 and 20 $\upmu \text{m}$, with the same elastic modulus for the entire lens capsule, E = 1.00 MPa, were analysed and compared.

#### Evaluation of the lens mechanical properties and geometry

Considering the elastic modulus values of the lens tissues (nucleus, cortex and capsule) to be homogeneous, a full factorial design was performed to analyse the effect of its magnitude on the ability of the lens to change its shape, i.e., the change in lens power (${\Delta L_P}$ – dioptres (D)), the change in lens thickness due to the nucleus ($\frac{\Delta L_T}{\Delta L_P}$ – %), the anterior and posterior radii of curvature ($\frac{\Delta r_a}{\Delta L_P}$ and $\frac{\Delta r_p}{\Delta L_P}$ – mm/D) and the lens thickness and diameter ($\frac{\Delta L_T}{\Delta L_P}$ and $\frac{\Delta \varnothing _L}{\Delta L_P}$ – mm/D). To do so, following the DoE methodology used in our previous work analysing the design of intraocular lenses (IOLs)^28,39^, a full factorial design with four factors (the elastic modulus values of the nucleus, cortex and anterior and posterior capsule) and five levels, i.e., $4^5 = 625$, was performed. The minimum and maximum level for the mechanical properties of the lens capsule and lens nucleus and cortex were obtained from literature^20–22,35,36^. Table 1 presents the elastic modulus levels of the factors analysed.

Furthermore, to observe the influence of the geometry, the analysis was performed for two lens geometries depending on age (17 and 29 y/o^29,30^), resulting in $625 \cdot 2=1250$ simulations. For these analyses, the distribution of the capsule thickness reported by Fincham^26^, indicated by the black line in Fig. 3, was considered.

**Table 1:** Material properties for the different levels (L) of the DoE.

Factors	Elastic modulus (kPa)				
	L1	L2	L3	L4	L5
Anterior capsule	100	300	500	700	1000
Posterior capsule	100	300	500	700	900
Cortex	0.75	1.50	2.25	3.00	3.75
Nucleus	0.10	0.20	0.30	0.40	0.50

Four factors and five levels were considered for two different geometries according to age (17 and 29 y/o).

## Results

In this section, the influences of the lens stiffness gradient is explained first. Then, the distribution of the capsule thickness on the ability of the lens to change its shape is analysed. Once both effects are numerically analysed, the lens mechanical properties for two different geometries corresponding to two different ages (17 and 29 y/o) are analysed following the design of experiments (DoE) methodology with a full factorial design^28^. To evaluate the ability of the lens to change its shape, the results are presented as a ratio between the variation of each biometric term and the change in lens power, i.e., the following ratios: $\frac{\Delta r_a}{\Delta L_P}$, $\frac{\Delta r_p}{\Delta L_P}$, $\frac{\Delta L_T}{\Delta L_P}$ and $\frac{\Delta \varnothing _L}{\Delta L_P}$. This was possible to perform because all analyses followed a linear response. Additionally, the maximum change in lens power ($\Delta L_P$) and the percentage of variation in the lens thickness due to the nucleus ($\frac{\Delta L_N}{\Delta L_T}$) were registered. All ratios and the maximum change in lens power are presented in absolute values. Therefore, a higher ratio between the anterior and posterior radius values ($\frac{\Delta r_a}{\Delta L_P}$, $\frac{\Delta r_p}{\Delta L_P}$) indicates a greater effect of the corresponding parameter on the focal change of the lens. Note that the ratios $\frac{\Delta r_a}{\Delta L_P}$ and $\frac{\Delta r_p}{\Delta L_P}$ can not be compared faithfully between them as their initial value is different and thus their absolute change affects differently in Eq. (2).

### Influence of the lens stiffness gradient

**Figure 4:**
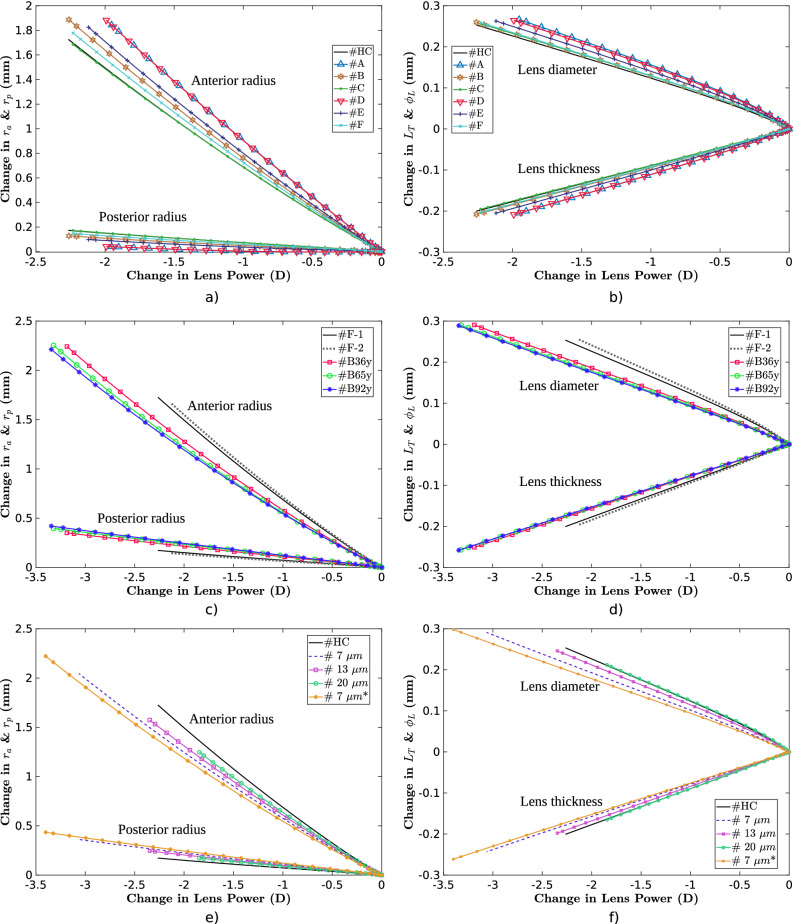
Change in the main biometric parameters of the lens for different case studies according to the focal change of the lens: anterior and posterior radius (**a**,**c**,**e**) and lens thickness and diameter (**b**,**d**,**f**). (**a**) and (**b**) depict the results for all stiffness gradient scenarios described in “Evaluation of the lens stiffness gradient” section; (**c**) and (**d**), the results for the five experimental distributions of the lens capsule thickness shown in Fig. 3; (**e**) and (**f**), the results for different uniform lens capsule thickness together with the reference scenario (#HC). All calculations were performed for the 29 y/o lens geometry and summarised in Table 2.

Figure 4a,b show the influence of the stiffness gradient on the dynamic optical and biometric lens measurements for the 29 y/o lens geometry. There is a notable difference between the homogeneous materials (nucleus and cortex) scenario #HC and scenario #A, especially in terms of the ${\Delta L_P}$, 2.26 against 1.95 D, an increase of 16 %. This is mainly produced by the ${\Delta r_p}$, which is 0.17 mm in scenario #HC against 0.03 mm for scenario #A, implying a disaccommodation amplitude of 0.42 D more for scenario #HC in the posterior surface term of Eq. (2). Although the change in the anterior surface is the main factor accountable for lens accommodation, ${\Delta r_a}$ was similar for both scenarios, 1.71 and 1.83 mm for scenarios #HC and #A, respectively, which implied a disaccommodation amplitude of only 0.11 D more for scenario #A.

**Table 2:** The results for the corresponding scenario, together with the corresponding section to which it belongs, gradient (G), thickness (T) or uniform thickness (UT), is described.

Scenario (section)	Factors					Change in the lens shape					
	CT	AC (MPa)	PC (MPa)	LC (kPa)	LN (kPa)	$\Delta L_P$ (D)	$\frac{\Delta L_N}{\Delta L_T}$ (%)	$\frac{\Delta r_a}{\Delta L_P}$ (mm/D)	$\frac{\Delta r_p}{\Delta L_P}$ (mm/D) $10^{-2}$	$\frac{\Delta L_T}{\Delta L_P}$ (mm/D) $10^{-2}$	$\frac{\Delta \varnothing _L}{\Delta L_P}$ (mm/D) $10^{-2}$
#HC, F-1 (G,T,TH)	F1	1.00	0.70	3.00	0.30	2.26	75.84	0.76	7.62	11.19	8.85
#A (G)	F1	1.00	0.70	E(FV)	E(FV)	1.95	71.45	0.94	2.04	13.63	10.50
#B (G)	F1	1.00	0.70	E(FV)	E(FV)	2.26	73.45	0.83	5.67	11.48	9.21
#C (G)	F1	1.00	0.70	E(FV)	E(FV)	2.23	74.99	0.75	7.73	11.48	8.75
#D (G)	F1	1.00	0.70	E(FV)	E(FV)	1.99	71.83	0.94	2.12	13.28	10.46
#E (G)	F1	1.00	0.70	E(FV)	E(FV)	2.11	73.65	0.86	4.63	12.39	9.67
#F (G)	F1	1.00	0.70	E(FV)	E(FV)	2.23	74.56	0.79	6.60	11.60	9.11
#F-2 (T)		1.00	0.70	3.00	0.30	2.13	75.81	0.78	6.80	11.98	9.21
#B-36y (T)		1.00	0.70	3.00	0.30	3.18	78.42	0.70	11.01	9.11	7.87
#B-65y (T)		1.00	0.70	3.00	0.30	3.32	80.25	0.67	11.94	8.73	7.73
#B-92y (T)		1.00	0.70	3.00	0.30	3.34	81.76	0.66	12.55	8.63	7.71
#7 $\upmu \text{m}$ (UT)		1.00	1.00	3.00	0.30	3.06	78.17	0.66	11.80	9.50	7.93
#10 $\upmu \text{m}$ (UT)		1.00	1.00	3.00	0.30	2.34	78.55	0.67	10.42	10.48	8.42
#20 $\upmu \text{m}$ (UT)		1.00	1.00	3.00	0.30	1.84	79.32	0.67	9.34	11.49	8.95
#7 $\upmu \text{m}$* (UT)		1.00	0.70	3.00	0.30	3.40	78.84	0.65	12.77	8.76	7.68

The corresponding factor for each scenario is described: the distribution of the capsule thickness (CT) for the gradient section. In turn, the section thickness describes itself, as do the Young’s modulus values of the anterior capsule (AC), posterior capsule (PC), lens cortex (LC) and lens nucleus (LN). Furthermore, the registered lens biometric measurements are presented: the maximum change in lens power ($\Delta L_P$), the variation in the lens thickness of the nucleus ($\frac{\Delta L_N}{\Delta L_T}$) and the following ratios: $\frac{\Delta r_a}{\Delta L_P}$, $\frac{\Delta r_p}{\Delta L_P}$, $\frac{\Delta L_T}{\Delta L_P}$, and $\frac{\Delta \varnothing _L}{\Delta L_P}$.

This difference in ${\Delta L_P}$ could be explained by an underestimation of the stiffness gradient. After all, the cortex and nucleus were not differentiated in the experimental study by Weeber et al.^22^. Thus, we designed four lens stiffness gradient scenarios with two different approaches (#B, #C, #E and #F). For all four scenarios, the results were similar to the homogeneous scenario (#HC) with uniform stiffness in the nucleus and cortex. Surprisingly, when the stiffness gradient starts in the nucleus (#C), the results are almost identical to those of the scenario with uniform stiffness (#HC).

Last, there was hardly any difference between scenarios #A and #D, which indicated the lower influence of the elastic modulus on the equatorial lens diameter. All analyses are summarised in Table 2.

### Influence of the lens capsule thickness

The influence of the distribution of the capsule thickness for the five cases analysed is shown in Fig. 4c,d. There is a statistically significant difference ($p_{value} < 0.05$) between the ratios obtained for all biometric terms with respect to the simulations performed with the distributions of the capsule thickness reported by Fincham^26^ and Barraquer et al.^27^.

The results obtained with the capsule thickness distribution reported by Barraquer et al.^27^ (referred to as Barraquer’s group hereinafter) presented a larger average change in lens power, 3.27 D against 2.18 D. For Barraquer’s^27^ group, a higher ratio in the posterior radius of curvature was presented, with an average value ($\frac{\Delta \overline{r_p}}{\Delta \overline{L_P}}$) of $11.80 \cdot 10^{-2}$ against $7.24 \cdot 10^{-2}$ mm/D for those obtained with the capsule thickness distribution reported by Fincham^26^ (referred to as Fincham’s group hereinafter). As a consequence, a lower ratio in the anterior radius was obtained, with an average value ($\frac{\Delta \overline{r_a}}{\Delta \overline{L_P}}$) of 0.68 against 0.77 mm/D.

The results for Barraquer’s^27^ group presented a lower ratio of lens thickness and diameter, with average values ($\frac{\Delta \overline{L_T}}{\Delta \overline{L_P}}$, $\frac{\Delta \overline{\varnothing _L}}{\Delta \overline{L_P}}$) of $7.78 \cdot 10^{-2}$ and $8.82 \cdot 10^{-2}$ mm/D, whereas for Fincham’s group, average values ($\frac{\Delta \overline{L_T}}{\Delta \overline{L_P}}$, $\frac{\Delta \overline{\varnothing _L}}{\Delta \overline{L_P}}$) of $9.05 \cdot 10^{-2}$ and $11.53 \cdot 10^{-2}$ mm/D were obtained, respectively. Moreover, the variation in lens nucleus with the change in lens thickness ($\frac{\Delta L_N}{\Delta L_T}$) was 80.14% as determined by Barraquer’s group, in comparison with 75.82% for Fincham’s^26^.

Regarding the case of a uniform capsule thickness, when the thickness is lower, the posterior radius of curvature exerts greater influence on the focal change of the lens, as displayed in Fig. 4e. The thinnest thickness (#7 $\upmu \text{m}$) of the lens capsule presented the highest lens focal change of 3.06 D, in comparison with 1.84 D for the thickest thickness (#20 $\upmu \text{m}$). Regarding the changes in lens thickness and diameter, as shown in Fig. 4f, the lower the uniform thickness is, the lower the ratio of the lens thickness and diameter. For the thinnest thickness (#7 $\upmu \text{m}$), $\frac{\Delta L_T}{\Delta L_P}$ and $\frac{\Delta \varnothing _L}{\Delta L_P}$ of $9.50 \cdot 10^{-2}$ and $7.93 \cdot 10^{-2}$ mm/D were obtained, respectively, whilst for the thickest thickness (#20 $\upmu \text{m}$), $\frac{\Delta L_T}{\Delta L_P}$ and $\frac{\Delta \varnothing _L}{\Delta L_P}$ of $11.49 \cdot 10^{-2}$ and $8.95 \cdot 10^{-2}$ mm/D were obtained, respectively. A similar case as the thinnest thickness (#7 $\upmu \text{m}$), but with an elastic modulus for the posterior capsule of 0.70 MPa, was also calculated (#7 $\upmu \text{m}$*), presenting a maximum change in lens power of 3.40 D. Interestingly, all cases present similar $\frac{\Delta L_N}{\Delta L_T}$ ratios of approximately 78%. All analyses are summarised in Table 2.

### Influence of the mechanical properties and age-related lens geometry on accommodation

After the 1, 250 simulations, a screening analysis was performed to remove data in which the change of the lens power increases. These cases corresponded to the lowest level of the elastic modulus of the cortex ($E=0.75$ kPa) and the highest levels of the nucleus ($E=0.50$ kPa) and the posterior capsule $(E=0.90$ MPa). This will be explained in the evaluation of the DoE. In total, 115 simulations were removed.

For the remaining simulations, 1, 135, a regression model with $R^2 > 99\%$ was designed for each response ($\Delta L_P$, $\frac{\Delta L_N}{\Delta L_T}$ $\frac{\Delta r_a}{\Delta L_P}$, $\frac{\Delta r_p}{\Delta L_P}$, $\frac{\Delta L_T}{\Delta L_P}$, and $\frac{\Delta \varnothing _L}{\Delta L_P}$) to evaluate the biomechanical effects of the factors of the DoE performed (*anterior capsule stiffness (AC), posterior capsule stiffness (PC), lens cortex stiffness (LC), lens nucleus stiffness (LN), and lens geometry depending on age (Age)*). The statistical model used to describe the results included up to second order terms.

**Figure 5:**
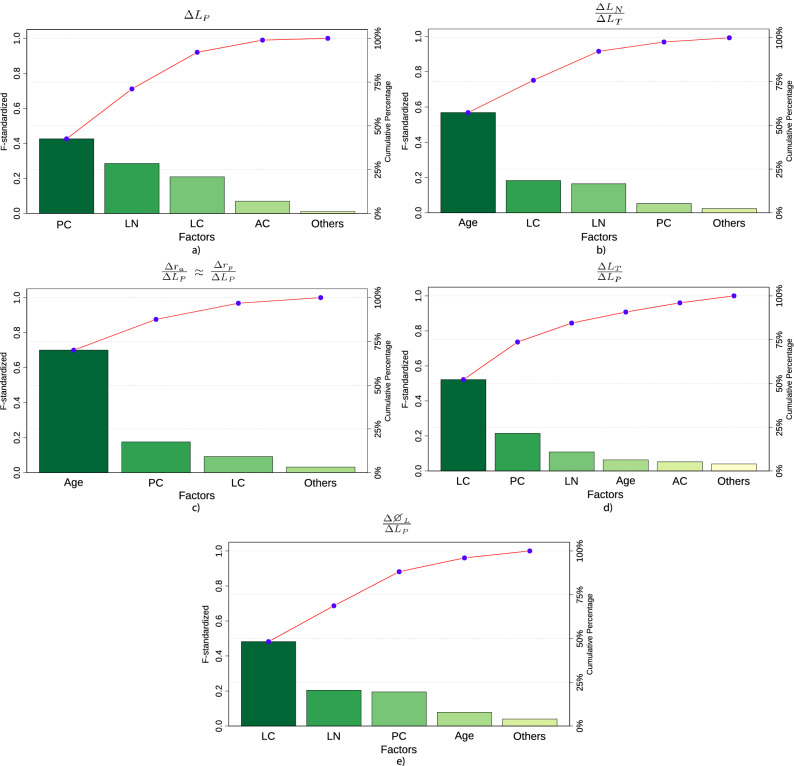
Pareto chart of the standardised effects on the six responses analysed: $\Delta L_P$ (**a**), $\frac{\Delta L_N}{\Delta L_T}$ (**b**), $\frac{\Delta r_a}{\Delta L_P}$ (**c**), $\frac{\Delta r_p}{\Delta L_P}$ (**c**), $\frac{\Delta L_T}{\Delta L_P}$ (**d**), and $\frac{\Delta \varnothing _L}{\Delta L_P}$ (**e**). As the results for the anterior and posterior radius were similar, a Pareto chart is used for both factors. Factors: the stiffness of the anterior capsule (AC), the posterior capsule (PC), lens cortex (LC) and lens nucleus (LN), and the age-related lens geometry (Age). Terms in the statistical model up to second order were included.

The influence of each factor on the responses analysed was evaluated by means of a Pareto analysis. The change in lens power ($\Delta L_P$) is mostly influenced by the stiffness of the posterior capsule (PC, 42.59%), the lens nucleus (LN, 28.49%) and the lens cortex (LC, 20.92%); see Fig. 5a. Other responses with respect to the age-related lens geometry were statistically significant, but their influences were lower than the stiffness of the PC, LN and LC.

The age-related lens geometry was the most influential factor (Age, 58.86%) on lens nucleus variation in the total change in lens thickness ($\frac{\Delta L_N}{\Delta L_T}$), as displayed in Fig. 5b, followed by the internal properties of the lens (LC, 18.95% and LN, 16.44%). The lens geometry depending on age (Age, 69.98%) was also the most relevant factor to the ratios $\frac{\Delta r_a}{\Delta L_P}$ and $\frac{\Delta r_p}{\Delta L_P}$; see Fig. 5c. As the initial anterior and posterior radii in the 17 y/o lens are lower, their variation produces a greater change in lens power; see Eq. (2). This explains the relevance of the age-related lens geometry to the ratios $\frac{\Delta r_a}{\Delta L_P}$ and $\frac{\Delta r_p}{\Delta L_P}$.

**Figure 6:**
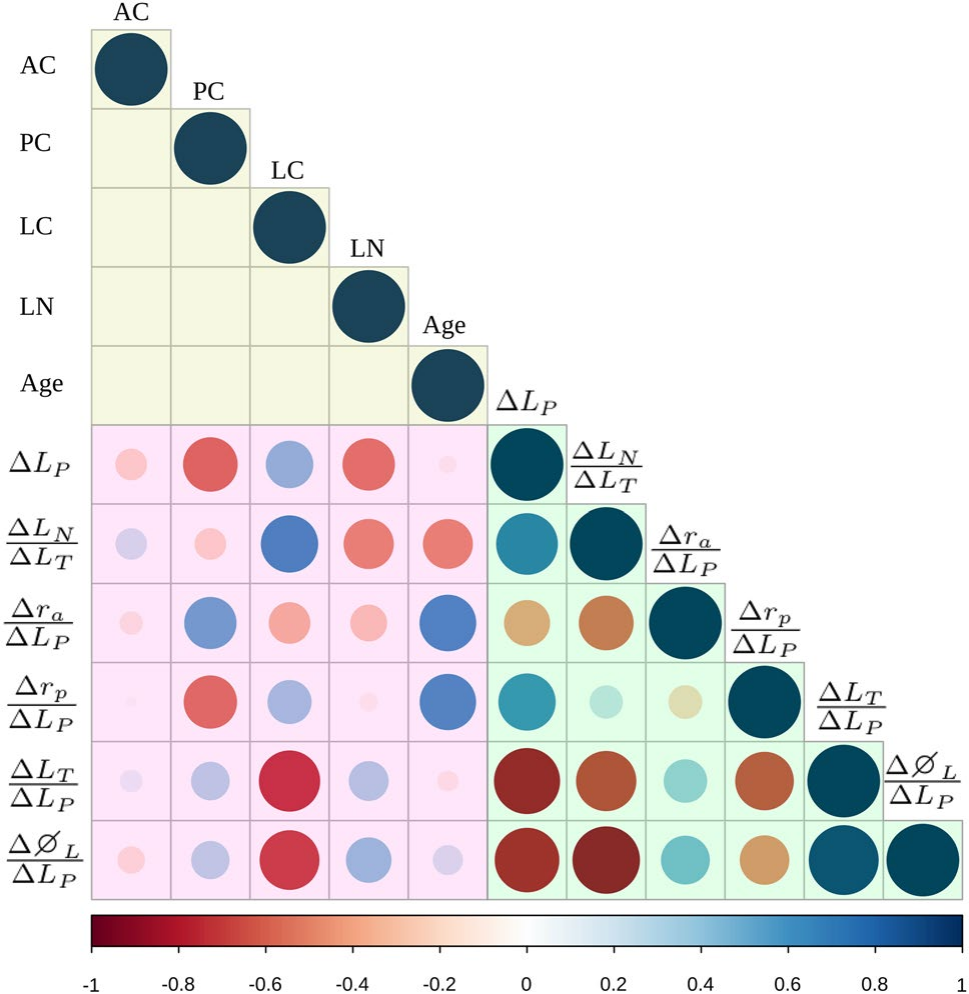
Pearson correlation matrix (full-level factorial design, $2 \cdot 5^4 = 1250$). Factors: stiffness of the anterior capsule (AC), posterior capsule (PC), lens cortex (LC) and lens nucleus (LN), and the lens geometry depending on age (Age). Level − 1 corresponds to 17 y/o and level 1 to 29 y/o). Biometric lens measurements: $\Delta L_P$, $\frac{\Delta L_N}{\Delta L_T}$ $\frac{\Delta r_a}{\Delta L_P}$, $\frac{\Delta r_p}{\Delta L_P}$, $\frac{\Delta L_T}{\Delta L_P}$, and $\frac{\Delta \varnothing _L}{\Delta L_P}$. The color of each circle depicts whether the linear correlation is direct (positive) or inverse (negative) (blueish palette, direct; reddish palette, inverse). The larger the circle diameter, the higher the correlation.

A similar trend occurs in the ratios $\frac{\Delta L_T}{\Delta L_P}$ and $\frac{\Delta \varnothing _L}{\Delta L_P}$; see Fig. 5d,e. The stiffness of the lens cortex is the most influential factor for both responses. To a lesser extent, the posterior capsule and lens nucleus also exert relevant influences on both responses. Further analysis based on the Pearson correlation matrix (see Fig. 6) supports these findings.

A stiffer lens cortex produced a greater change in the lens power ($\Delta L_P$); see Fig. 6. Moreover, the posterior capsule and the lens cortex were confirmed to be strongly inversely correlated with the change in lens power. Surprisingly, $\frac{\Delta r_a}{\Delta L_P}$ and $\frac{\Delta r_p}{\Delta L_P}$ presented a high correlation with the posterior capsule, but not with the anterior capsule, which confirms that the posterior capsule governs the change in lens power. Regarding the change in lens thickness due to the nucleus ($\frac{\Delta L_N}{\Delta L_T}$), the greater the difference in stiffness between the nucleus and cortex, the larger the change in $\frac{\Delta L_N}{\Delta L_T}$ is. For the younger geometry, the $\frac{\Delta L_N}{\Delta L_T}$ was higher.

A stiffer posterior capsule produced a higher ratio $\frac{\Delta r_a}{\Delta L_P}$ and a lower ratio $\frac{\Delta r_p}{\Delta L_P}$. As mentioned, the 29 y/o lens (value 1 in Fig. 6) requires a greater change in the anterior and posterior radii to produce the same change in lens power (strong direct correlation with $\frac{\Delta r_a}{\Delta L_P}$ and $\frac{\Delta r_p}{\Delta L_P}$).

The stiffness of the lens cortex was strongly inversely correlated with the ratios $\frac{\Delta L_T}{\Delta L_P}$ and $\frac{\Delta \varnothing _L}{\Delta L_P}$, which indicates that a stiffer lens cortex produces a lower change in the ratios $\frac{\Delta L_T}{\Delta L_P}$ and $\frac{\Delta \varnothing _L}{\Delta L_P}$. The posterior capsule and lens nucleus exhibited direct correlation with $\frac{\Delta L_T}{\Delta L_P}$ and $\frac{\Delta \varnothing _L}{\Delta L_P}$. The $\frac{\Delta L_T}{\Delta L_P}$ ratio presented a slight direct correlation with the anterior capsule and a slight inverse correlation with the age-related lens geometry. Contrary to this, the $\frac{\Delta \varnothing _L}{\Delta L_P}$ ratio presented the opposite behaviour as described for the $\frac{\Delta L_T}{\Delta L_P}$.

Regarding the correlation between the lens biometric terms, there was a strong direct correlation between the change in lens power ($\Delta L_P$) and the $\frac{\Delta r_p}{\Delta L_P}$ ratio, whereas there was a strong inverse correlation between $\frac{\Delta L_T}{\Delta L_P}$, $\frac{\Delta \varnothing _L}{\Delta L_P}$ and the change in lens power ($\Delta L_P$).

There was an inverse correlation between the $\frac{\Delta r_a}{\Delta L_P}$ and $\frac{\Delta r_p}{\Delta L_P}$ ratios, a strong inverse correlation between $\frac{\Delta r_p}{\Delta L_P}$ and $\frac{\Delta L_T}{\Delta L_P}$ and a strong direct correlation between $\frac{\Delta L_T}{\Delta L_P}$ and $\frac{\Delta \varnothing _L}{\Delta L_P}$. Main effect and interaction plots were analysed to confirm the consistency of the correlation analysis.

## Discussion

The results in this study provide essential data regarding how the crystalline lens changes its shape depending on different lens factors such as the stiffness gradient of the lens^21,22^, the distribution of the capsule thickness^26,27^, the mechanical properties of the lens nucleus, cortex and anterior and posterior capsules in young subjects^35,36^, and the influence of two different lens geometries depending on age (17 and 29 y/o)^29^.

The stiffness gradient of the lens was evaluated using different possible scenarios for a young adult subject based on experimental data^22^. There was a significant difference in the posterior radius response between scenario #A, with a stiffness gradient (see Fig. 4a,b and Table 2), and a scenario with homogeneous materials (scenario #HC). Nevertheless, it is true that the stiffness gradient might be understudied, as the mechanical properties of the nucleus and cortex were not individually characterised due to the high complexity of taking these measurements. When smoothing the stiffness gradient, there was similarity between the results presented (see scenarios #B, #C, #E and #F in Figs. 3a, 4b and Table 2). As observed in the study, the difference in stiffness between the nucleus and cortex is crucial to how the lens changes its shape. The greater the difference is, the more significant the effect of the posterior and anterior surface, and thus the higher the change in lens power, as presented throughout the study. Based on this statement, the ideal case is the scenario with homogeneous materials (#HC), where the difference between the stiffness of the nucleus and cortex is the greatest and most abrupt. Thus, the closer the gradient is to the homogeneous case, the greater the focal change of the lens. Therefore, we consider that the assumption of modelling the nucleus and cortex with homogeneous behaviour is appropriate for a theoretical approach.

Regarding the capsule thickness distribution, five different distributions, divided into Fincham^26^ and Barraquer et al.^27^ groups, and four different uniform thicknesses were evaluated. The main difference was that for Barraquer’s^27^ group, there is a decrease in thickness in the posterior capsule, as shown in Fig. 3. This decrease entails a higher lens focus and diminished changes in lens thickness and diameter, mainly produced by the increased changes in the posterior radius and lens nucleus. The trend was confirmed by the homogeneous scenarios; see Table 2. A thinner capsule thickness produces a similar effect to that of a capsule with a lower elastic modulus.

The use of numerical models along with advanced statistical tools (i.e., full factorial analysis) allows the study of the impacts of lens factors (material behaviour of the anterior capsule, posterior capsule, lens cortex and nucleus, and the age-related lens geometry) on the ability of the lens to change its shape (change in lens power (${\Delta L_P}$), percentage of the lens nucleus in the change in the lens thickness ($\frac{\Delta L_N}{\Delta L_T}$), and the following ratios which reproduce how the shape of the lens changes: $\frac{\Delta r_a}{\Delta L_P}$, $\frac{\Delta r_p}{\Delta L_P}$, $\frac{\Delta L_T}{\Delta L_P}$, and $\frac{\Delta \varnothing _L}{\Delta L_P}$). Based on the presented results, the mechanical properties of the posterior capsule, lens cortex and lens nucleus are the most important factors affecting the ability of the lens to change its shape. The age-related lens geometry and the stiffness of the anterior capsule were also statistically significant, but their influences were lower.

Individually, the analysed factors differently influenced the ability of the lens to change its shape; see Fig. 6. A greater stiffness in the anterior capsule produced a lower change in lens power. It also presented a weak direct correlation with the lens thickness and a weak inverse correlation with the lens diameter. The posterior capsule presented a strong inverse correlation with the change in lens power and the posterior radius. A stiffer lens cortex implied a greater change in lens power and a higher ratio with respect to the posterior radius. The stiffness of the cortex was strongly inversely correlated with the thickness and diameter ratios. The stiffness of the lens nucleus presented the opposite behaviour as that of the cortex, which confirmed the strong influence of the difference in stiffness between both observations in the elastic gradient section. Furthermore, the screening analysis performed in the DoE where 115 simulations were removed showed that the lens would lose its accommodative properties if the stiffness ratio between the nucleus and cortex would be close to 1.00, which could explain some causes of presbyopia. The stiffness of the lens nucleus might have presented a weaker correlation than the cortex due to the lower variation in the levels of the DoE performed.

Finally, the 17 y/o lens geometry presented a higher change in lens power. The change in the lens geometry was strongly and directly correlated with the anterior and posterior radius ratios because the changes in the anterior and posterior radii differ depending on the initial values in the accommodated state. A variation in the anterior radius from 6.00 to 8.00 mm does not influence the same focal change as the variation from 8.00 to 10.00 mm; see Eq. (2). Furthermore, the lens thickness ratio was lower in the 29 y/o lens geometry, whilst the lens diameter ratio was larger.

One of the limitations of this study is that contrary to the works of Manns et al.^13^ and Augusteyn et al.^14^, the force exerted to stretch the lens could not be compared with experimental data. This result was due to two factors: first, it would have involved the characterisation of all relevant tissues (ciliary body and sclera), and second, the exact geometry involved in these experimental tests. Nevertheless, with the essential data provided in this study, any change in lens shape for any hominid species, including humans, could be reproduced, which validates the model. One weakness is that the study was focused on healthy crystalline lenses (17–29 y/o). It would be interesting to broaden the scope of this research to include pathological eyes, with cataracts or presbyopia, of older adults.

To ease the understanding of the objective of the study, the thick lens formula has been used, see Eq. (2), instead of a GRIN optical model. The authors have checked that the pattern results of the study is the same with a four-surface shell model^40^ and the use of Eq. (2) with an equivalent refractive index. The authors also have checked the consistency of the study observing the change in curvature of the posterior and anterior radius nucleus. Moreover, Van Sompel et al.^7^ also showed for the 29 y/o subject that the relative lens accommodative amplitude appears to be unaltered using an uniform or GRIN optical model. The group will focus on validating a GRIN optical model for the crystalline lens in future investigations.

Unlike other numerical papers that modelled the zonules with few “wires”, we have modelled them in order to provide continuity in the displacement field of the lens. Their design was set according to experimental images^34^. Contrary to other studies, we evaluated the main biometric lens parameters which explain its change in shape ($\frac{\Delta r_a}{\Delta L_P}$, $\frac{\Delta r_p}{\Delta L_P}$, $\frac{\Delta L_T}{\Delta L_P}$, $\frac{\Delta \varnothing _L}{\Delta L_P}$ and $\frac{\Delta L_N}{\Delta L_T}$), and interestingly, most simulations prove that the $\frac{\Delta L_N}{\Delta L_T}$ ratio was approximately 80%, as Brown^31^ experimentally reported. Like Van Sompel et al.^7^, we observed that the lens geometry has an important role with respect to the change in lens focus. However, the change in lens focus is overly complex since it depends on so many factors, including capsule thickness, mechanical properties of all parts of the lens, geometry, etc., that it is difficult to highlight one over the others. The situation becomes even more complicated in accommodation, which is produced not only by focal changes of the lens but also by lens movement. This study provides compelling evidence that the stiffness values of the posterior capsule, lens cortex and nucleus are the most influential factors with respect to the ability of the lens to change its shape.

This work has been made possible by the emergence of new quality experimental studies, which enables the design of more complex and realistic numerical models. This study has demonstrated how lens focus is influenced by the main lens mechanical properties, allowing for a slightly improved understanding of the accommodation process. We hope to illuminate the mechanisms of the accommodation field, helping manufacturers and researchers find better solutions for dysfunctional lens conditions such as presbyopia and cataracts. Furthermore, our group will be focused on how presbyopia progresses according to the mechanical properties of age-related lens.
